# Evaluation of methods for detecting conversion events in gene clusters

**DOI:** 10.1186/1471-2105-12-S1-S45

**Published:** 2011-02-15

**Authors:** Giltae Song, Chih-Hao Hsu, Cathy Riemer, Webb Miller

**Affiliations:** 1 Center for Comparative Genomics and Bioinformatics, 506 Wartik Lab, Pennsylvania State University, University Park, PA 16802, USA; 2 Computational Biology Branch, National Center for Biotechnology Information, National Library of Medicine, National Institutes of Health (NIH), Bethesda, MD, USA

## Abstract

**Background:**

Gene clusters are genetically important, but their analysis poses significant computational challenges. One of the major reasons for these difficulties is gene conversion among the duplicated regions of the cluster, which can obscure their true relationships. Many computational methods for detecting gene conversion events have been released, but their performance has not been assessed for wide deployment in evolutionary history studies due to a lack of accurate evaluation methods.

**Results:**

We designed a new method that simulates gene cluster evolution, including large-scale events of duplication, deletion, and conversion as well as small mutations. We used this simulation data to evaluate several different programs for detecting gene conversion events.

**Conclusions:**

Our evaluation identifies strengths and weaknesses of several methods for detecting gene conversion, which can contribute to more accurate analysis of gene cluster evolution.

## Background

Gene clusters are genomic regions that comprise multiple similar copies in close proximity, generated by duplication from a common ancestral segment. These duplicated segments often contain genes, but we also include non-genic regions in this study. In the human genome, gene clusters are of special interest to researchers because of their genetic and molecular biological importance. Many clusters are implicated in diseases having a genetic component, such as cancer and immune system disorders.

To understand how gene clusters are involved in these diseases, inferring their evolutionary histories is very helpful. Constructing a phylogenetic tree or a multiple sequence alignment is the most common initial step when studying gene cluster evolution. Both of these approaches assume that all of the positions in a duplicated copy will show similar divergences from the original segment, so we expect only one phylogeny for a given set of DNA sequences [1] and only one multiple alignment for all of their orthologous sequences [2]. However, existing phylogenetic tree construction methods produce different tree topologies depending on which part of each duplicated segment is taken as the input data, while multiple sequence alignments sometimes align non-orthologous parts of the sequences.

**Figure 1:**
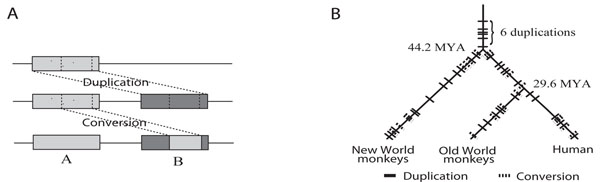
**Depiction of a conversion event and an example of simulation**. (A) Depiction of a conversion event. First, a duplication copies region *A* to *B*, and *A* and *B* begin to diverge through substitutions and other small-scale events. Second, part of *B* is overwritten by its homologous segment from *A* in a conversion event. As a result, the converted segment becomes more similar than the other parts of *A* and *B*. (B) An example of simulation, with three sequences that mimic those of New World monkeys (NWM), Old World monkeys (OWM), and humans. NWM split from the human lineage  million years ago (MYA), and have about 89% DNA sequence similarity with human.OWM separated  MYA, with about 93% similarity to the human sequence. Randomly applied substitutions simulate the divergence rates among these three clades, starting from an ancestral sequence (see main text). The bold and dotted lines represent duplication and conversion events, respectively, and the location of each line indicates the time of the event. In this example, the simulated human lineage has a total of 18 duplication and 8 conversion events from the starting ancestral sequence.

One of the major causes of these difficulties is the occurrence of so-called “gene conversion” events. A conversion event occurs between two paralogous (genic or non-genic) segments that were formed by a previous duplication. During such an event, part of one segment is copied to its homologous location in the other segment, overwriting that portion of the homologous sequence. This makes the target sequence a mosaic of sub-segments with varying divergences from the source sequence (Figure 1A). Conversion events are typically caused by DNA double-strand breaks or by a double Holliday-junction dissolution mechanism [3].

Many computational methods for detecting gene conversion have been developed. When we consider methods requiring only DNA sequence data, they are classified in two main categories. The first includes phylogenetic-based methods, which identify gene conversions by finding breakpoints that change the tree topology, using either the maximum parsimony principle [4], maximum likelihood method [5-8], or Bayesian methods such as hidden Markov models [9-13]. The second category contains methods based on sequence similarity, which search for segments of unusually high similarity within two homologous regions [14-22]. RDP3 [23] integrated 10 existing methods, including [9,14,15,18,19]. Note that methods which require additional information beyond the DNA sequences, such as the true duplication history or polymorphism data, are excluded in this study.

Although many programs for detecting gene conversion have been released, and some have wider currency than others, little is known about their relative merits and demerits, especially in gene clusters. Several studies have evaluated the performance of the major methods. [18] suggested using simulation data with varying levels of recombination, genetic diversity, and mutation rate. [24] performed an evaluation with real datasets where the “true” conversions were already known. More recently, [20] and [25] compared conversion detection methods on simulated data with higher conversion rates than previous studies, even including multiple overlapping conversions in the same region. Other studies (e.g. [26-36]) have developed DNA-evolution simulators that incorporate conversion events, which could be used to evaluate conversion detection methods. All of these results can provide useful information for investigating gene conversions when a set of short homologous sequences is already given; however they do not clearly reveal which conversion detection method is best for large-scale evolutionary studies (viz., those that include duplication, deletion, inversion, and conversion events), because they focus only on microevolutionary processes for purposes of population genetics studies. Although sequence evolution simulators that do include large-scale events such as insertion and deletion have been developed in other studies (e.g. [37-41]), they do not consider conversion events. Hence none of these previous evaluations or simulators adequately accounts for gene clusters containing multiple sets of homologous segments of varying length. We have developed a method to evaluate the performance of conversion detection programs with regard to untangling gene cluster histories. We compared several of the major programs with a new one from our lab [22] using simulation data that resembles gene clusters containing many unknown homologous sequence pairs generated by repeated duplications.

## Methods

### Simulation of gene cluster evolution, including conversion

The simulation process starts with a 200 kb human DNA sequence that contains no duplications (i.e., when it is aligned to itself using the LASTZ alignment program [42] with default parameters, no self-alignments corresponding to paralogous sequence pairs are generated). The sequence is modified with large-scale events such as duplications, deletions, and conversions, interspersed with small-scale mutations. The latter are simulated based on the HKY substitution model [43], while the distribution of duplication and deletion events was obtained from 53 human gene clusters from [44] in which duplications were detected using the Monte Carlo Markov Chain method. Our parameters for generating these events, such as the length of the duplicated or deleted region, the space between the original copy and the new one, and the orientation of the new one, are modeled from these empirical distributions. For gene conversions we used software from [22], which detects conversions genome-wide, and ran it on the human genome to obtain our distribution for modeling conversion events.

Each conversion event must occur between two paralogous sequences, which were formed by previous duplications. However, conversion events are allowed to occur orthogonally to duplication events, so that any duplicated segment can undergo conversion with any other segment. For example, if region *A* is copied to region *B* by one duplication and *B* is copied to *C* by another duplication, then (*A*, *C*) are paralogous as well as (*A*, *B*) and (*B*, *C*), so conversion can happen between *A* and *C*. We also consider partial copying of previously duplicated segments (e.g., *C* can be copied from part of *B*), and furthermore, several previously duplicated tandem segments (or a portion thereof) can be duplicated again together, as a single region. These factors make the paralogous relationships more complicated. We have implemented a program to generate a true sequence alignment according to specified duplication and deletion histories, so that we can keep track of all paralogous pairs.

Based on the empirical parameter distributions, a series of duplication, deletion, and conversion events is generated. (Note that for the purposes of our simulation, we combine duplications and deletions into a single category, so a particular “duplication” in what follows might actually refer to a deletion.) To simulate the evolution of gene clusters at various complexity levels, several sets of cluster data were generated that experienced different numbers of events. The order of the large-scale events is decided randomly, and these are interspersed with nucleotide substitutions. In our simulation, the times of duplication and conversion events are assigned according to a uniform distribution along each species lineage. Figure 1B shows an example of a simulation dataset indicating the time of each duplication and conversion event. The other properties of each event are chosen according to their respective empirical distributions. For example, when simulating a conversion event, a pair of paralogous segments is chosen at random from all true local self-alignments formed by the preceding duplications, and then the location and length of the converted region within those segments are determined using the applicable distributions.

We simulated gene cluster evolution for three primate clades: humans, Old World monkeys (OWM), and New World monkeys (NWM), starting from a common ancestral sequence. NWM splits from the human lineage first, followed by OWM (Figure 1B). The NWM and human sequences are roughly 89% similar, while OWM has about 93% similarity to human [44]. By mutating these sequences based on the HKY model, we can get divergence rates that are quite similar to the real genome data. However, we cannot assume that the entire sequence is under neutral evolution. In order to design a more realistic evolutionary model, purifying selection in regions such as protein-coding exons and other functional elements should be considered. According to [45], about 5% of the human genome is covered by conserved elements, and their lengths average around 100-120 bp in a geometric distribution. We model this by choosing regions randomly from such a distribution until they cover 5% of the starting ancestral sequence, and set them as the conserved regions in this simulation. We assume that they evolve about 30% slower than the neutral sites on average (but this parameter can be changed easily).

All of the properties taken into account when designing the simulation datasets are summarized in Table 1.

**Table 1:** Parameters for simulating evolutionary events in gene clusters

Event Type	Properties
Duplication	Number of duplications Location of duplication Length of duplicated region Space between the original copy and the new one Orientation of the new duplicated copy

Deletion	Number of deletions Location of deletion Length of deleted region

Conversion	Number of conversions Length of the two paralogous sequences Space between the two paralogous sequences Relative orientation of the two paralogous sequences Location of the two paralogous sequences Location of converted region Length of converted region Direction of conversion

Small-scale mutation	HKY substitution model Divergence rates between species

Purifying selection	Locations of elements under purifying selection Lengths of elements Mutation rate of elements

Other	Timing of large-scale events in each species lineage

### Preparation of data for running conversion detection programs

The pipeline from Hsu et al. [22] includes a procedure for identifying orthologs for each pair of paralogous sequences, given the corresponding gene cluster sequences from multiple species. However, most software for detecting gene conversions (e.g., GENECONV [14] and recHMM [13]) requires a multiple alignment of homologous sequences from multiple species as input. In order to run such programs on a gene cluster, we need to identify all sets of homologous sequences and construct the multiple alignment in advance. The evaluation of alternative methods for doing this is beyond the scope of this study. Since the only requirement for running the pipeline from Hsu et al. is to provide sequences as input data, we used their entire package including its orthology detection method. For the other programs, we used “true” multiple alignments of the true homologous sequences.

**Figure 2:**
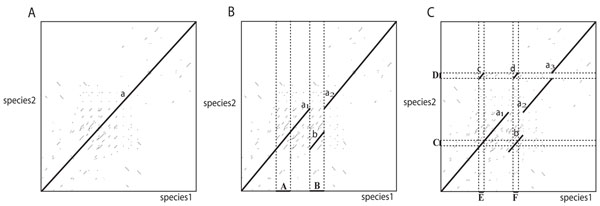
**An example of tracking true orthologs during simulation of duplications**. (A) The bold line shows a long orthologous alignment *a* between two species immediately after they split from their ancestral sequence. (B) A duplication copying *A* to *B* in species1 splits *a* into two alignments *a*_1_ and *a*_2_, and generates an additional orthologous alignment *b*. (C) A subsequent duplication copies *C* to *D* in species2, splitting *a*_2_ and generating *c* and *d*, for a total of six orthologous alignment segments.

The true multiple alignments are obtained as follows. First, the true orthologous sequences are identified in each simulation dataset using the true evolutionary history from the simulation process. For example, suppose we have two species called *species*1 and *species*2. Right after these split, their sequences align perfectly as a long orthologous alignment *a*, shown as a bold line in Figure 2A. If a region *A* in *species*1 is copied to *B* by a duplication event, then the orthologous alignment is split into two alignments *a*_1_ and *a*_2_ and an additional orthologous alignment *b* is also formed in the new duplicated region between them, as in Figure 2B. Subsequent duplication events can generate multiple additional alignments, such as the one in Figure 2C that adds two alignments, *c* and *d*. By keeping track of these alignments while applying the evolutionary events, the true orthologous sequences can be identified. Second, the true self-alignments (where a sequence aligns to itself) are also computed based on the series of evolutionary events in each species. New self-alignments are added by duplications, and they can be split by subsequent events. As each simulated event occurs, new alignments and changes in old alignment boundaries are tracked so that the final true self-alignments can be obtained. Finally, a multiple alignment is generated for each set consisting of a pair of paralogous sequences in one species and all of their orthologous sequences in the other species. The paralogous sequences correspond to a self-alignment, so they can be obtained from the true self-alignments already computed. Their orthologs are selected from the true orthologous alignments. For example, suppose we have a self-alignment of segments *C* and *D* in *species*2 from Figure 2C, and their orthologs *E* and *F* are identified from the true inter-species alignments; then a multiple alignment of *C*, *D*, *E*, and *F* is constructed.

## Results and Discussion

We chose four methods of detecting gene conversion that are able to identify multiple breakpoints given a set of homologous sequences: Hsu’s [22], GENECONV [14], RDP3 [23], and recHMM [13]. We ran these programs on simulation data generated using various parameters for the complexity of the evolutionary processes. One parameter is the number of duplication and conversion events. First, *d* duplications are simulated before the split of NWM and human, for *d* = {3, 6, 9, 12}. Next, an additional *d* duplications are applied to each species before the split of OWM and human, and *c* conversion events are also simulated in the paralogous sequences of each species, where *c* = {2, 4, 6, 8} respectively. Finally, *d* duplications and *c* conversion events are applied to all species after the split of OWM and human. In the end, we get three sequences (NWM, OWM, and human), each having experienced 3*d* duplications and 2*c* conversion events, plus interspersed small-scale mutations. Each such dataset comprises approximately 50-200 pairs of paralogous segments, and we generated five replications for each of the four settings of (*d*, *c*). Another parameter is the selection model for applying the nucleotide mutations. One uses only neutral evolution, while the other includes both neutral evolution and purifying selection. The conserved sites under purifying selection are assigned as described in the Methods section. The resulting datasets are available from our website, at http://www.bx.psu.edu/miller_lab/.

**Figure 3:**
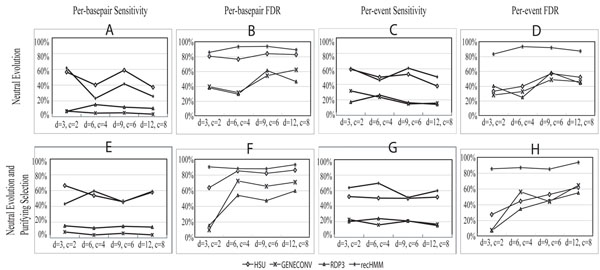
**Summary of the detection results**. 3*d* duplication and 2*c* conversion events were simulated in each dataset, for *d* = {3, 6, 9, 12} and *c* = {2, 4, 6, 8} respectively. *d* duplications were simulated before the split of NWM and human, then an additional *d* duplications plus *c* conversion events were applied in each species up to the split of OWM and human. Finally, *d* duplications and *c* conversion events were applied to all species after the split of OWM and human. In A-D only neutral evolution is modeled, whereas both neutral evolution and purifying selection are included in E-H. Each point represents the mean of five replications (fewer for recHMM). (A),(E) The sensitivity of the methods for detecting converted basepairs. (B),(F) The false discovery rate (FDR) for the per-basepair detection. (C),(G) The sensitivity for detecting the existence of gene conversion events. (D),(H) The FDR for the existence detection.

All of the conversion detection programs were then run (using their default settings) on each of the generated datasets, except that recHMM was not run on all of the replications because it is very slow. The recHMM method may take a day or longer to process a dataset with complex settings, while the others typically finish within an hour, even for complicated cases. Figure 3 compares the performance of the four detection methods. Panels A-D show the results obtained for datasets simulating only neutral evolution, while E-H are for datasets modeling both neutral evolution and purifying selection. In A we calculated the fraction of converted basepairs that were detected correctly. On average, Hsu’s method detected about 51% of the true converted positions, GENECONV 5%, RDP3 11%, and recHMM 38%. We also compared the false discovery rates (FDR) by calculating the fraction of called basepairs that were incorrect. As panel B shows, Hsu’s method had an FDR of about 82%, GENECONV 52%, RDP3 44%, and recHMM 91%. In this comparison of the accuracy of conversion boundaries, Hsu’s method shows higher sensitivity than the others, while RDP3 has the fewest false positives.

We also measured the performance of the four programs for detecting the existence of gene conversion, i.e. how many pairs of paralogous sequences which experienced conversion are detected as such, even if the exact endpoints of the conversion are not identified correctly. In Figure 3C, the sensitivity of Hsu’s method by this measure is quite similar to panel A, but the FDR (panel D) drops by 33% compared to panel B. For GENECONV, RDP3, and recHMM, the sensitivities increase by 16%, 7%, and 16% and the FDRs decrease by 9%, 2%, and 2% respectively. A possible reason for this may be that Hsu’s method tends to detect the boundaries of converted regions as wider than their true extent, while the other three methods tend to identify boundaries that are too narrow.

When we model purifying selection in addition to neutral evolution, the four performance metrics are quite similar to the simpler model, except that the sensitivity of recHMM improves (Figure 3E-H). Overall, for both selection models, the sensitivity seems to be roughly inversely proportional to the FDR. In terms of the detection sensitivity, Hsu’s method and recHMM show the best performance. When we consider both sensitivity and FDR, Hsu’s method outperforms the others for detecting the existence of gene conversion events, but has a relatively high FDR when identifying the extent of the converted regions.

The relative performance of the four methods in terms of sensitivity and FDR is mostly independent of the number of events. However, there is an overall tendency for the sensitivity to drop and the FDR to rise as the evolutionary complexity increases, e.g. when there are more events. There are exceptions to this, for example the sensitivity increases significantly between the 2nd and 3rd columns in Figure 3A for Hsu’s method and recHMM. These apparent anomalies may be due to the influence of other parameters from Table 1 on the complexity, since we have only a few replications and statistical variation in those other parameters could have a sizeable impact.

## Conclusions

We evaluated four gene conversion detection methods using simulated DNA sequence datasets for gene clusters that were generated by an evolutionary model. We found that Hsu’s method and recHMM showed the highest sensitivity for detecting both the existence and extent of conversion events, but their FDRs are higher than those of GENECONV and RDP3. Interestingly, the FDR of Hsu’s method drops drastically when detecting only the existence of conversion in a paralogous pair, as opposed to the exact boundaries of the converted region. If we consider both the detection power and the false positives, Hsu’s method would be the most recommended for gene cluster evolution studies. However, it may need additional careful post-processing for filtering false positive errors.

Our evaluation method is still in its infancy, although it already provides useful information. Our next short-term plan is to extend our simulation tool for a larger number of species and to add more conversion detection methods that were excluded from this study (e.g., because they are only suitable for a single breakpoint detection or conversion existence test). That may require developing a post-processing pipeline to extend each method to generate multiple breakpoints. We also plan to compare our evaluation results with those obtained using previous DNA-evolution simulators that model only micro-scale events. Another future goal is to design a more realistic evolutionary model for gene clusters by reflecting additional evolutionary events such as codon and amino acid substitution, positive selection, insertion of interspersed repeats, and structural variations such as inversions and small-scale deletions and insertions. By modeling evolutionary processes in gene clusters more accurately, our ongoing efforts can contribute to improving software for the analysis of gene cluster evolution.

## Competing interests

The authors declare that they have no competing interests.

## Authors' contributions

GS and CH designed the method. GS implemented the simulation program and analyzed the results. WM initiated, supervised, and coordinated the work. GS and CR wrote and finalized the manuscript, and all authors read and approved it.
